# Isolation and characterization of *Aspergillus niger* NBC001 underlying suppression against *Heterodera glycines*

**DOI:** 10.1038/s41598-018-37827-6

**Published:** 2019-01-24

**Authors:** Na Jin, Shi-Ming Liu, Huan Peng, Wen-Kun Huang, Ling-An Kong, Yu-Huan Wu, Yong-Pan Chen, Feng-Yong Ge, Heng Jian, De-Liang Peng

**Affiliations:** 1 State Key Laboratory for Biology of Plant Diseases and Insect Pests, Institute of Plant Protection, Chinese Academy of Agricultural Sciences, Beijing, China; 2 Key Laboratory of Plant Pathology of Ministry of Agriculture, College of Plant Protection, China Agricultural University, Beijing, China

## Abstract

*Heterodera glycines* is the most pervasive soybean pests worldwide. Biocontrol provides a strategy to sustainably control nematodes. In this study, 22 fungal isolates were obtained and identified from cysts of *Heterodera* spp. Among them, *Aspergillus niger* NBC001 showed high nematicidal activity against *H*. *glycines*. The 2-fold dilution of NBC001 culture filtrate caused 89% mortality of second-stage juveniles and inhibited more than 98% of egg hatching i*n vitro*. In both pot and field experiments, the numbers of *H*. *glycines* cysts in soybean seedlings dressed with the the 5-fold concentrated culture filtrate of NBC001 were significantly reduced by 43% and 28%, respectively. In addition, application of NBC001 remarkably reduced the penetration of nematodes into the roots. Histochemical and fluorometric staining analyses indicate that application of NBC001 stimulated hydrogen peroxide activity in the roots and triggered callose deposition in the leaves and roots. Transcription of the *PR1a* and *EREBP* genes in the salicylic acid and ethylene signaling pathways was upregulated in soybean plants treated with NBC001. However, the application of concentrated culture filtrate of NBC001 had no significant impacts on the soil microbial community based on next generation DNA sequencing technology. In summary, NBC001 may be a good biocontrol agent against *H*. *glycines* via stimulation of the immunity/defense of the plant host.

## Introduction

Soybean cyst nematodes (SCN, *Heterodera glycines* Ichinohe) are one of the most economically important pests causing considerable damage to soybean [*Glycine max* (L.) Merr.]^1^. SCN causes more than $120 million dollars in yield losses annually in China alone^2,3^. Chemical nematicides are the efficient ways to control *H*. *glycines*, but their application is being increasingly limited or banned due to their toxicity to humans and ecosystems^4^. Therefore, alternative safe methods are urgently required to control *H*. *glycines*. The use of biocontrol agents to suppress *H*. *glycines* could provide an alternative strategy to sustainably control *H*. *glycines* because of their eco-friendliness and general non-toxicity to humans.

Previously assessed biological control agents for managing *H*. *glycines* include nematophagous fungi, endoparasitic fungi, fungi that produce antibiotic substances, plant-growth-regulatory bacteria, and fungi or bacteria that induce systemic resistance (ISR) and plant defense responses in soybean^5^. Among these, *Aspergillus* species are very common in agricultural and non-agricultural soils, and are known to be lethal to a variety of plant-parasitic nematodes^6^. This fungus could significantly reduce the population of plant-parasitic nematodes in the soil and enhance the yield of plants^7,8^. Other biological control agents control nematodes by either producing antibiotic substances, parasitizing nematodes or inducing plant resistance to plant-parasitic nematodes. However, *A*. *niger* control nematodes by all the three ways. Oxalic acid, citric acid and undetermined compounds with molecule weight greater than 8000 have previously been reported to be nematicidal metabolites of *A*. *niger*^7–9^. The producation of NH_3_, HCN and siderophores may also contribute to the suppression of plant-parasitic nematodes^10,11^. In addition, *A*. *niger* has also been found to parasitize nematodes. Research has shown that the fungus grows and sporulates well on eggs, juveniles and adult females of root-knot nematodes^11^. Another mechanism of *A*. *niger* against plant-parasitic nematodes is that *A*. *niger* could induce the plant resistance. Li *et al*. (2011) reported that *A*. *niger* could reduce the nematode populations and promote the growth of tomato plants by increasing the activities of defense enzymes (phenylalanine ammonia, polyphenol oxidase, peroxidase, superoxide dismutase and catalase)^12^. This finding indicated that *A*. *niger* could markedly induce the resistance of tomatoes to plant-parasitic nematodes. However, the possibility that *A*. *niger* interacts with plant tissues and induces host plant resistance to pathogens has seldom been studied.

Most studies have focused on the effects of biocontrol microorganisms on the pathogens but neglected the responses of the host plant. Plants are able to recognize microbe and tailor their defense responses. These responses include the accumulation of phytoalexins, reinforcement of plant cell walls, production of reactive oxygen species (ROS) and callose, and synthesis of pathogenesis-related (PR) proteins, such as chitinases and glucanases^13^. Pathogen-induced systemic resistance, known as systemic acquired resistance (SAR), is commonly associated with salicylic acid (SA) and pathogenesis-related protein 1 (PR-1), while beneficial microorganisms trigger induced systemic resistance (ISR), which is often dependent on the jasmonate (JA) and/or ethylene (ET) signaling pathways with the concomitant induction of LOX and EREBP^14^. However, some ISR inducers also appear to activate the SA-dependent pathway, indicating that different signaling pathways may be operated when ISR is elicited^15^.

Soil microbes play a predominant role in agro-ecosystem. They can affect soil nutrient cycling, organic matter formation and decomposition and soil structure, thus influencing ecosystem stability^16^. The application of biocontrol agents could perturb the composition and function of soil microbial communities^17^. This might affect the soil quality for sustainable plant production and the promotion of plant health. Therefore, it is necessary to assess the effects of biocontrol agents on the soil microbial communities before they are used extensively.

In this study, we isolated strain *A*. *niger* NBC001 from *Heterodera* spp. cysts, and investigated the biocontrol efficacy of *A*. *niger* NBC001 against *H*. *glycines* on soybean. The ability of the ISR activity in soybean to suppress *H*. *glycines* infection was studied through the application of *A*. *niger* strain. In addition, we applied next generation sequencing (NGS) technology to compare the changes in soil microorganisms in response to the application of NBC001 and provide basic insights on risk assessment before the future commercialization of NBC001. These results will provide new strategies for the practical management of *H*. *glycines* and new insights into the mechanisms underlying the biocontrol processes of *A*. *niger*.

## Results

### Isolation and identification of fungi

Twenty-two fungal isolates were obtained from cysts of *Heterodera* spp. collected from the Xuchang District, Henan, China and Østfold, Norway. The fungal isolates represented ten genera, and *Mortierella alpina* was the most prominent species (Table 1). Thirteen representative isolates were selected for further study.

**Table 1:** Fungal isolates obtained from *Heterodera* spp.

Accession number	Fungal species	Sampling locations	Isolate from
NBC001	*Aspergillus niger*	Østfold, Norway	*H*. *avenae*
NBC002	*Aspergillus flavus*	Østfold, Norway	*H*. *avenae*
NBC003	*Aspergillus fumigatus*	Østfold, Norway	*H*. *avenae*
NBC004	*Periconia macrospinosa*	Østfold, Norway	*H*. *avenae*
NBC006	*Penicillium sp*.	Østfold, Norway	*H*. *avenae*
NBC008	*Penicillium oxalicum*	Østfold, Norway	*H*. *avenae*
NBC010	*Alternaria alternata*	Østfold, Norway	*H*. *avenae*
NBC011	*Asperigillus fumigatus*	Østfold, Norway	*H*. *avenae*
NBC012	*Penicillium oxalicum*	Østfold, Norway	*H*. *avenae*
NBC013	*Doratomyces sp*.	Østfold, Norway	*H*. *avenae*
NBC014	*Microdochium bolleyi*	Østfold, Norway	*H*. *avenae*
NBC015	*Plectosphaerella cucumerina*	Xu Chang district, Henan, China	*H*. *filipjevi*
NBC016	*Trichoderma brevicompactum*	Xu Chang district, Henan, China	*H*. *filipjevi*
NBC017	*Mortierella alpina*	Xu Chang district, Henan, China	*H*. *filipjevi*
NBC018	*Mortierella alpina*	Xu Chang district, Henan, China	*H*. *filipjevi*
NBC019	*Mortierella alpina*	Xu Chang district, Henan, China	*H*. *filipjevi*
NBC020	*Mortierella alpina*	Xu Chang district, Henan, China	*H*. *filipjevi*
NBC021	*Mortierella alpina*	Xu Chang district, Henan, China	*H*. *filipjevi*
NBC022	*Mortierella alpina*	Xu Chang district, Henan, China	*H*. *filipjevi*
NBC023	*Mortierella alpina*	Xu Chang district, Henan, China	*H*. *filipjevi*
NBC024	*Mortierella alpina*	Xu Chang district, Henan, China	*H*. *filipjevi*
NBC025	*Fusarium oxyporum*	Xu Chang district, Henan, China	*H*. *filipjevi*

### Effects of the isolated fungi on the J2s mortality of *H*. *glycines in vitro*

**Figure 1:**
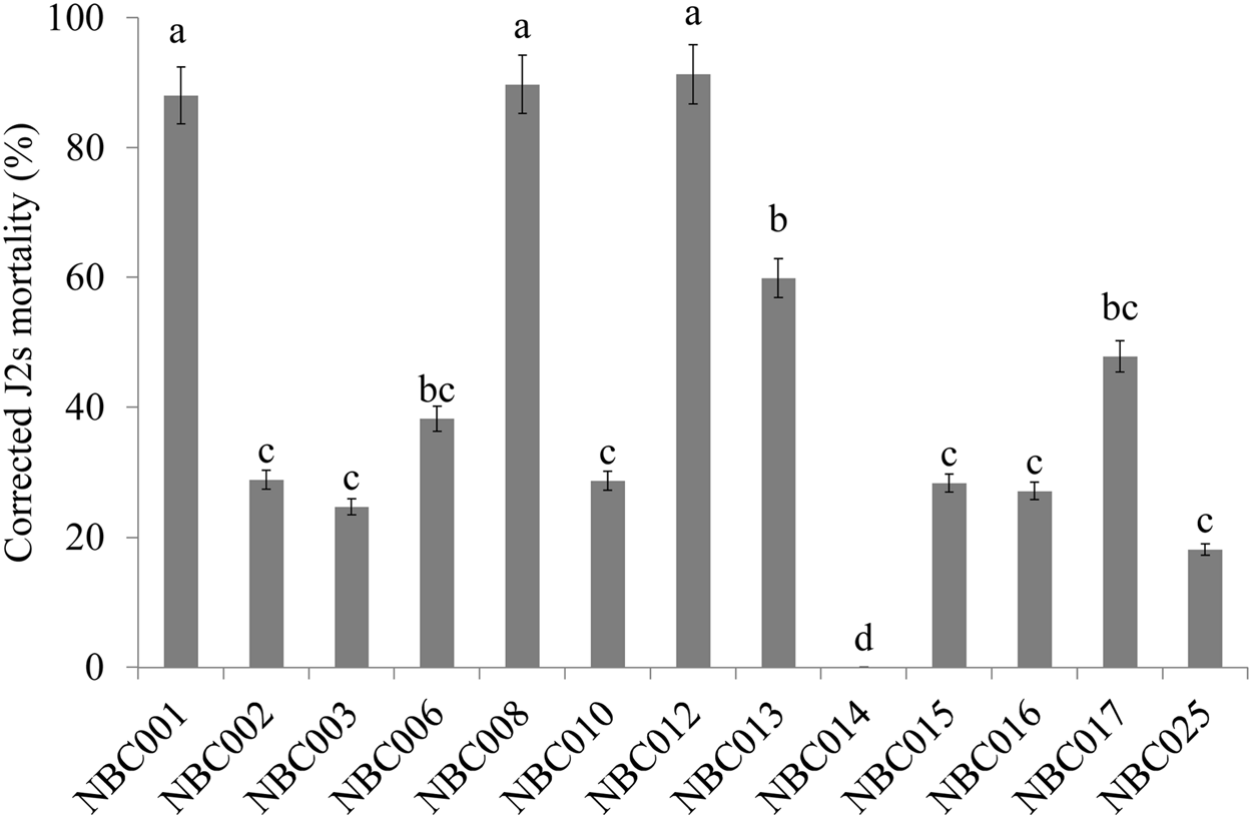
Effects of isolated fungi on the J2s mortality of *H*. *glycines*. Corrected J2s mortalities of *H*. *glycines* after exposure to the culture filtrate of the isolated fungi. The error bars represent the standard deviation. The different letters on the bar represent significant differences at the 0.05 level based on Tukey’s multiple comparison test.

Thirteen fungal isolates were used to test for nematicidal activity *in vitro*. Although the nematicidal activity varied among the fungal species and isolates, almost of all the isolates could kill the J2s of *H*. *glycines* to different degrees (Fig. 1). Among them, the treatments of 2-fold dilutions of the culture filtrates of *Aspergillus niger* NBC001, *Penicillium oxalicum* NBC008 and *Penicillium oxalicum* NBC012 resulted in significantly higher corrected J2s mortalities of *H*. *glycines* than others, which were 88%, 90% and 92%, respectively (*P* < 0.05). NBC001 was selected for further study because some *Aspergillus* species have already been studied for their biocontrol potentials against plant parasitic nematodes^18^.

### Effects of NBC001 on the J2s mortality and egg hatchability of *H*. *glycines in vitro*

**Figure 2:**
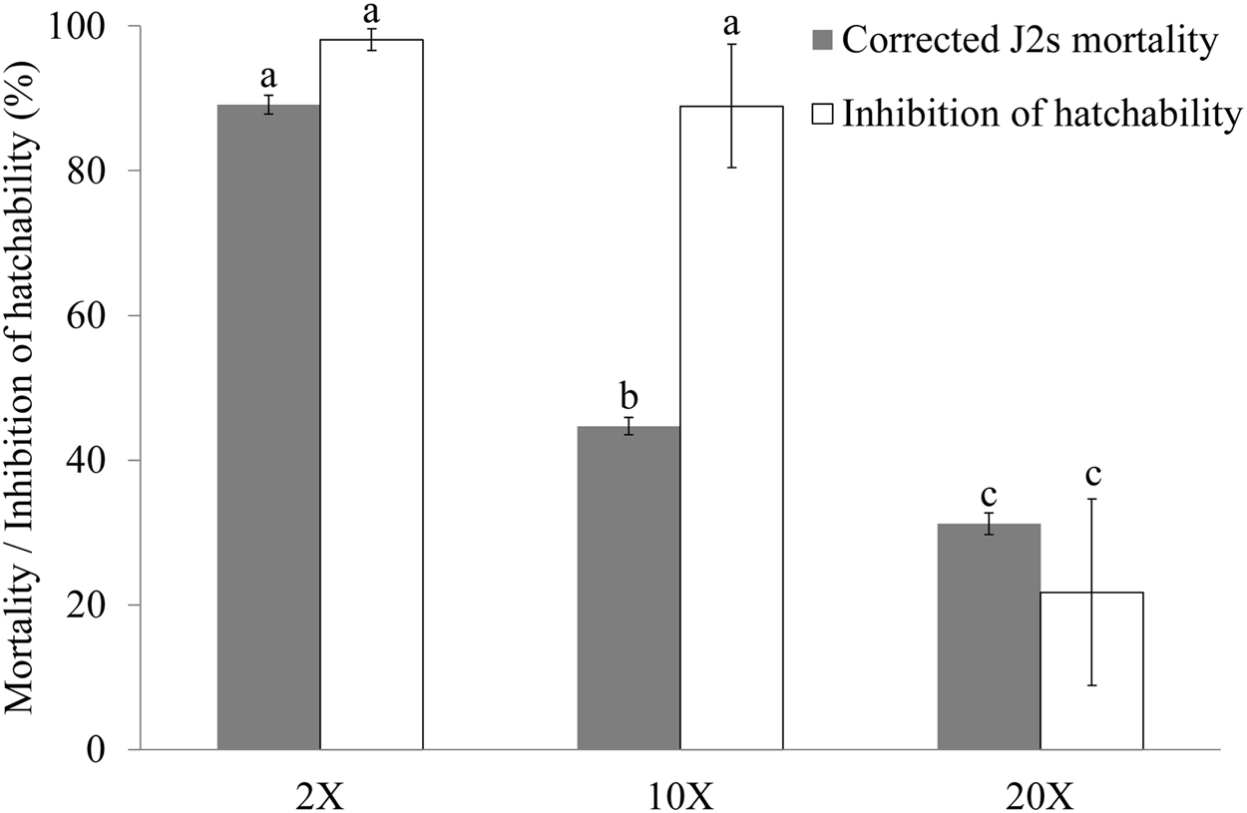
Effect of *Aspergillus niger* NBC001 on the J2s mortality and hatchability of *H*. *glycines*. Corrected J2s mortality and inhibition of the hatchability of *H*. *glycines* after exposure to various concentrations of *Aspergillus niger* NBC001 culture filtrate. The error bars represent standard deviation. The different letters on each bar represent significant differences at the 0.05 level based on Tukey’s multiple comparison test.

As shown in Fig. 2, all of the culture filtrate of NBC001 at various concentrations had significant effects on the J2s mortality and egg hatchability of *H*. *glycines*. The 2 × , 10 × , and 20 × dilutions of the culture filtrate of NBC001 caused 89%, 45% and 31% corrected J2s mortality of *H*. *glycines*, respectively. The egg hatchability in all the treatments of the 2 × , 10 × , and 20 × dilutions of the culture filtrate of NBC001 was significantly lower than those in the control, and the correspondingly relative inhibitions of hatchability were 98%, 89%, 22%, respectively. These results indicate that NBC001 showed high nematicidal activity against *H*. *glycines*.

### Efficacy of NBC001 against *H*. *glycines*

**Figure 3:**
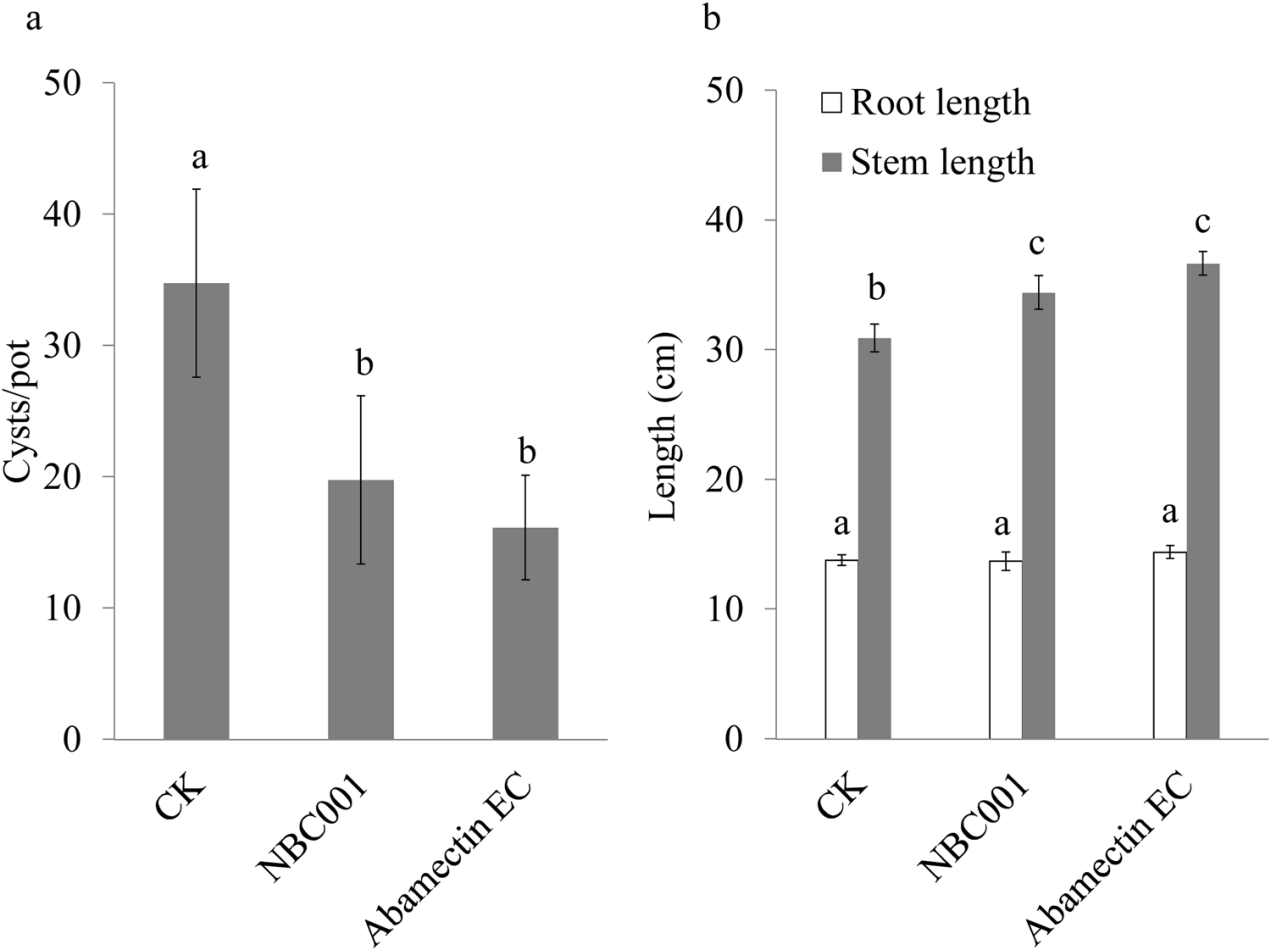
Effect of *Aspergillus niger* NBC001 on *H*. *glycine*s control in the pot. The numbers of *H*. *glycines* cysts in the soil (**a**) and stem and root lengths of soybean (**b**) at 30 d post-transplantation in the pot. The error bars represent the standard deviation. The different letters on each bar represent significant differences at the 0.05 level based on Tukey’s multiple comparison test.

**Figure 4:**
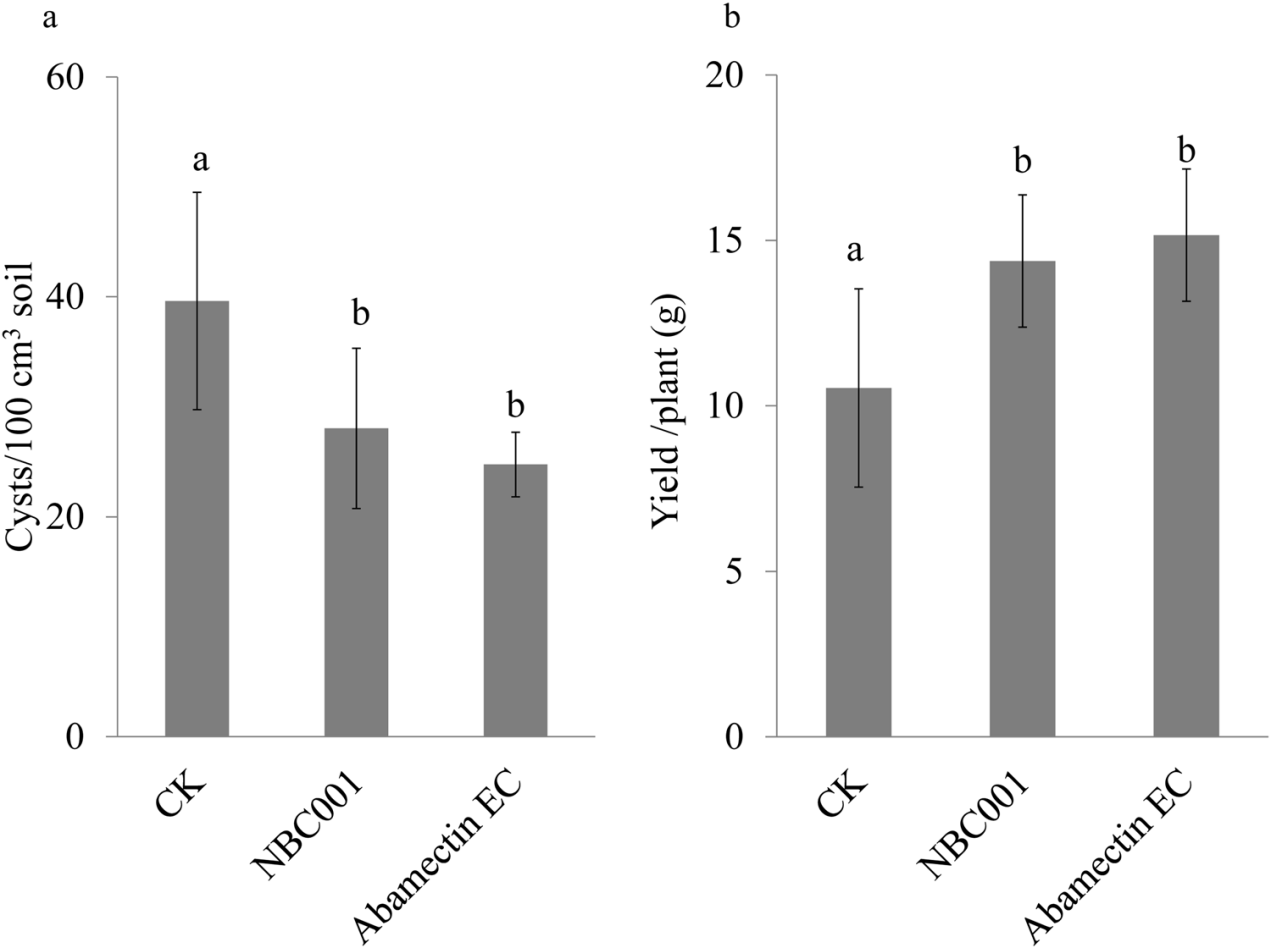
Effect of *Aspergillus niger* NBC001 on *H*. *glycine*s control in the field. The numbers of *H*. *glycines* cysts in the soil at 90 d post-transplantation in the field. The error bars represent the standard deviation. The different letters on each bar represent significant differences at the 0.05 level based on Tukey’s multiple comparison test.

The efficacy of the 5-fold concentrated culture filtrate of NBC001 against *H*. *glycines* was tested in both the pot and the field (Figs 3 and 4). In the pot, the numbers of *H*. *glycines* cysts in the NBC001 treatment was significantly reduced by 43% at 30 d post-transplantation compared to that in the control (Fig. 3a, *P* < 0.05), but those cyst numbers in the NBC001 treatment showed no significant differences from those in the abamectin EC treatment (53% of reduction compared to the control). The stem lengths of the soybeans in both the NBC001 treatment and the abamectin EC treatment were significantly increased by 10% and 16%, respectively, compared to that in the control (Fig. 3b, *P* < 0.05). However, the root lengths of the soybean did not show significant differences among any of the treatments (Fig. 3b, *P* > 0.05). In the field, the NBC001 and abamectin EC treatments significantly reduced the numbers of *H*. *glycines* cysts by 28% and 38% at 90 d post-transplantation, and increased the yield by 37% and 44.8%, respectively (Fig. 4a,b, *P* < 0.05). These results indicate that soybean seedlings dressed with the culture filtrate of NBC001 could control *H*. *glycines* in both the pot and the field, and the control efficacy of NBC001 was not significantly different from that of the commercial pesticide abamectin EC.

### Effect of NBC001 on nematode development in soybean roots

**Figure 5:**
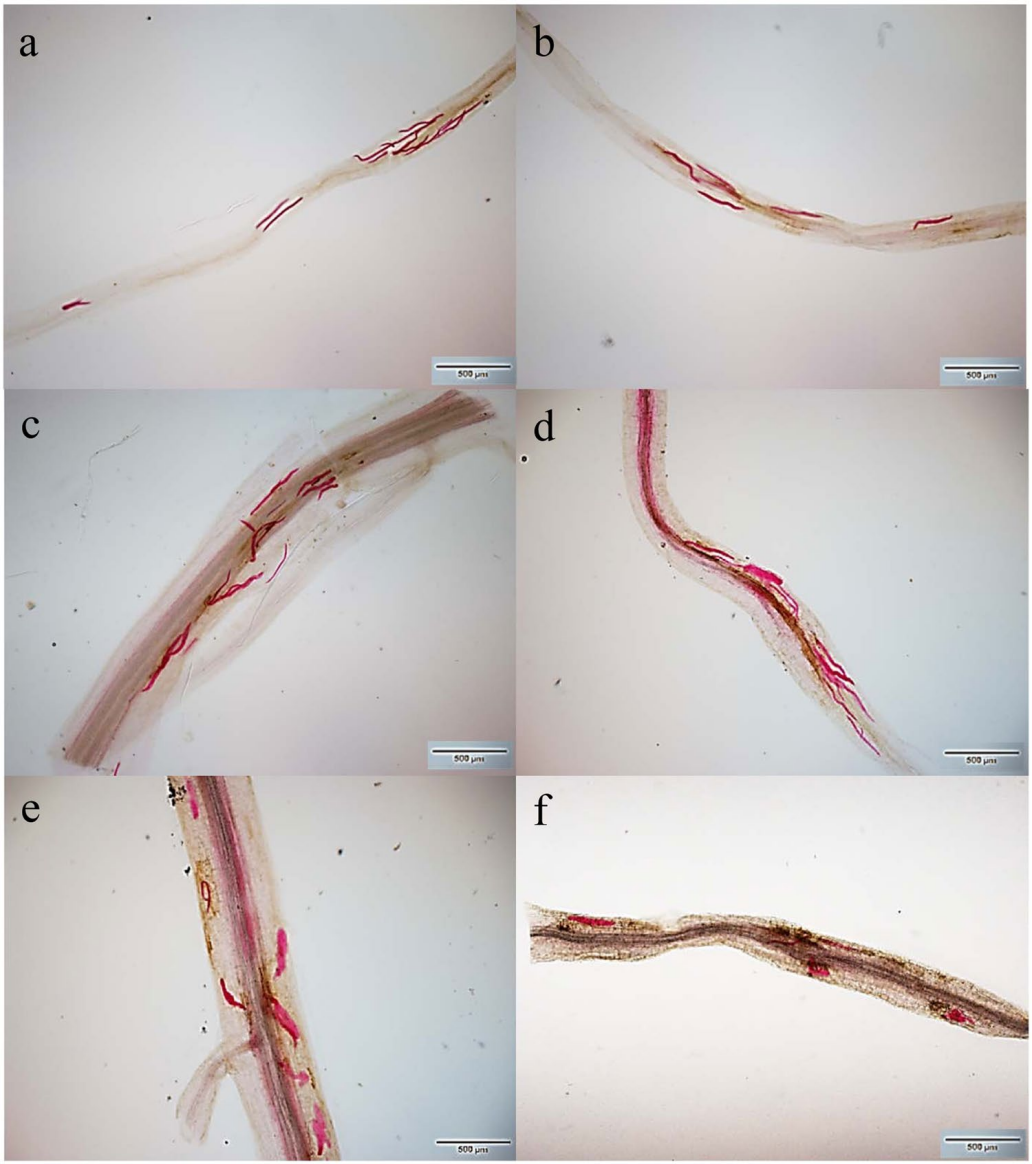
Penetration and development of *H*. *glycines* in soybean roots. Observations of juveniles of different developmental stages in the control soybean root group at 3 DPI (**a**), 7 DPI (**c**), and 14 DPI (**e**). Observations of juveniles of different developmental stages in the NBC001 treated soybean root group at 3 DPI (**b**), 7 DPI (**d**), and 14 DPI (**f**). Scale bars represent 500 μm.

At 3, 7 and 14 d post-inoculation, significantly fewer juveniles were observed in the roots of the NBC001 treatment than in the roots of the control (Table 2, Fig. 5, *P* < 0.05). At 3 d post-inoculation, the juveniles in the soybean roots were all at the J2 stage (Fig. 5a,b). At 7 d post inoculation, significantly fewer J2 juveniles were observed in the NBC001-treated soybean roots than that in the control soybean roots (*P* < 0.05), but the numbers of J3 juveniles among the treatments were not significantly different (Table 2, Fig. 5c,d, *P* > 0.05). At 14 d post-inoculation, the numbers of J2 and J3 juveniles in the NBC001 treatment were significantly lower than those in the control roots (*P* < 0.05), but the numbers of J4 juveniles among the treatments were not significantly different (Table 2, Fig. 5e,f, *P* > 0.05). All the results indicate that the application of NBC001 significantly reduced the nematode penetration rates but did not affect nematode’s development inside the roots.

**Table 2:** Numbers of *H*. *glycines* at different developmental stages in the soybean roots.

Time*	Treatments	Numbers of different stages of juveniles			Numbers of total juveniles
		J2	J3	J4	
3 DPI	CK	143 ± 41a	0 ± 0	0 ± 0	143 ± 41b
	NBC001	92 ± 33b	0 ± 0	0 ± 0	92 ± 33c
7 DPI	CK	132 ± 25a	64 ± 31a	0 ± 0	196 ± 53a
	NBC001	93 ± 33b	62 ± 28a	0 ± 0	155 ± 60b
14 DPI	CK	56 ± 5c	60 ± 4a	24 ± 1a	140 ± 8b
	NBC001	32 ± 5d	32 ± 5b	16 ± 4a	80 ± 11c

The different letters on each bar represent significant differences at the 0.05 level based on Tukey’s multiple comparison test.

*DPI, days post-inoculation. Mean ± standard deviation of the mean from four soybean root replicates.

J2, second-stage juvenile; J3, third-stage juvenile; J4, fourth-stage juvenile.

### Effects of NBC001 on callose deposition and ROS production in soybean

**Figure 6:**
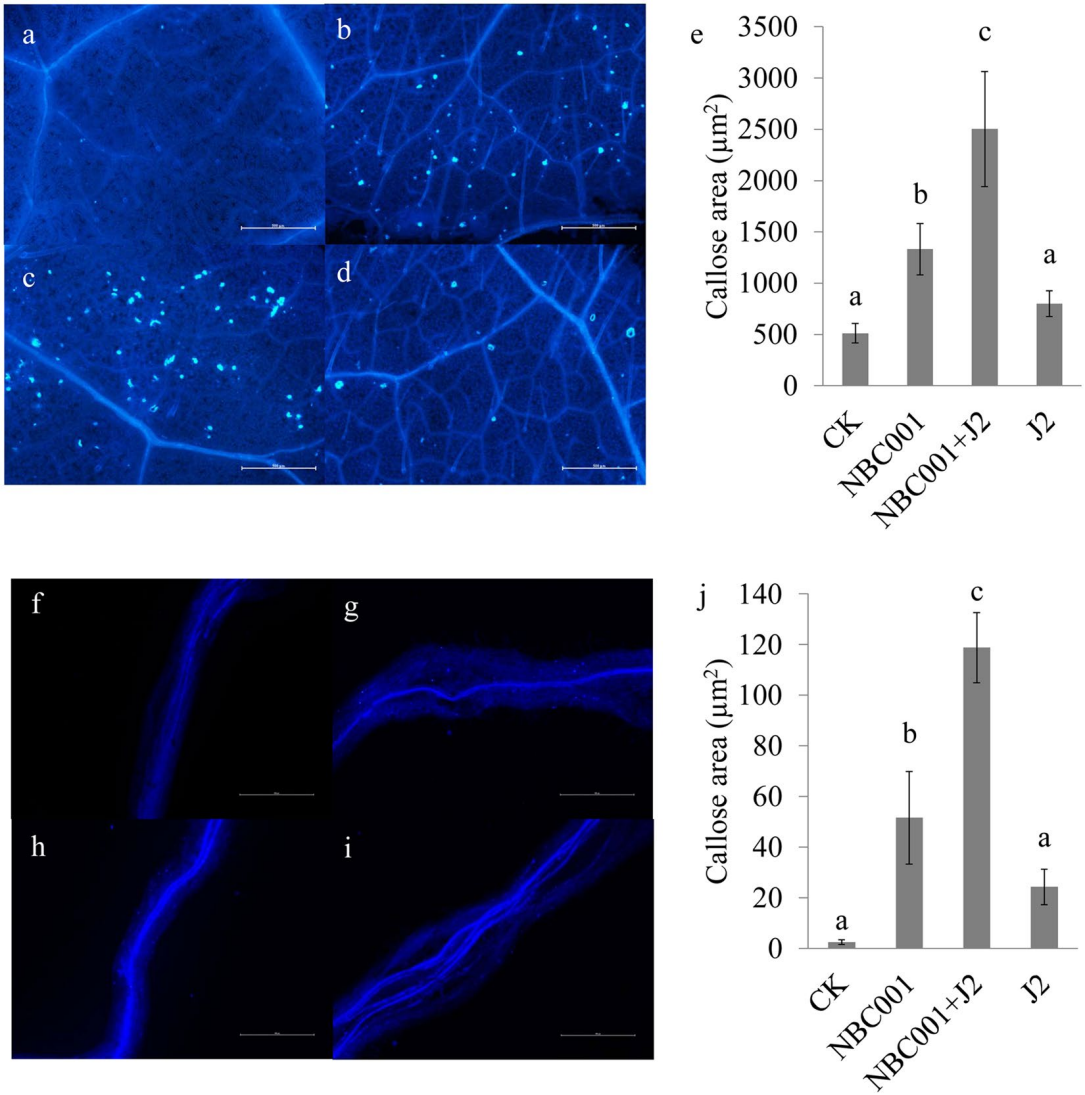
Histochemical analysis of cell wall reinforcement in the leaves and roots of soybean at 24 h post-inoculation. (**a**,**f**) Control, (**b**,**g**) NBC001 treatment, (**c**,**h**) NBC001 + J2 treatment, and (**d**,**i**) J2 treatment. Scale bars represent 500 μm. (**e**,**j**) Callose deposits were quantified using ImagePro 5.0 software. The values are the mean callose deposits area ± standard deviation from 20 pieces of leaves (diameter, 1 cm) or the roots from five plants.

We hypothesized that NBC001 could activate the soybean defense responses. Therefore, callose deposition and ROS production in soybean were evaluated at 24 h post-inoculation. Treatment of the soybean seedlings with the culture filtrate of NBC001 led to an increase in the callose deposition in both soybean leaves and roots compared to that resulting from the control treatment (Fig. 6). Measurement of the total areas of callose deposition spots from 20 randomly selected leaf discs or roots from each treatment revealed that a larger area of callose deposition was significantly displayed in both the NBC001 treatment and the NBC001 and J2 treatments compared to those in the J2 treatment and control (Fig. 6e,j, *P* < 0.05).

**Figure 7:**
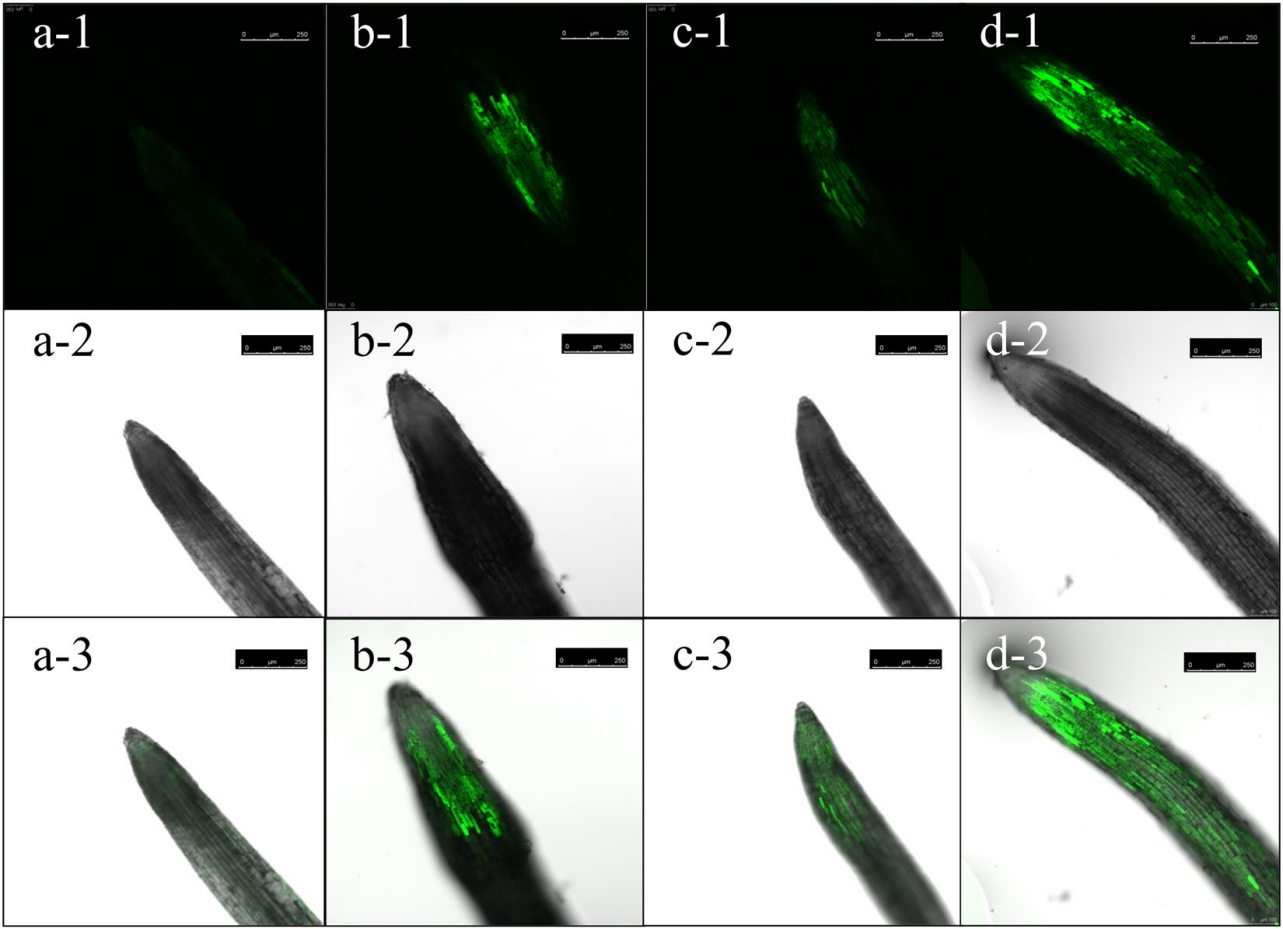
Histochemical analysis of the production of reactive oxygen species in the leaves of soybean at 24 h post-inoculation. (**a**) Control, (**b**) NBC001 treatment, (**c**) J2 treatment, and (**d**) NBC001 + J2 treatment. Scale bars represent 250 μm.

We labeled soybean roots with 5-(and-6)-chloromethyl-2′, 7′- dichlorodihydrofluorescein diacetate (CM-H2DCFDA), which fluoresces when activated by ROS in living cells. We used confocal laser scanning fluorescence microscopy to monitor the fluorescence in roots labeled with CM-H2DCFDA at 24 h post-innoculation. As shown in Fig. 7, the extent of CM-H2DCFDA fluorescence was increased in roots treated with both NBC001 and J2 and treated with only NBC001 compared to those in the roots treated with J2 or the control. These results indicate that treating soybean seedlings with the culture filtrate of NBC001 triggers ROS production in the soybean roots.

### Expression analysis of plant defense-related genes

**Figure 8:**
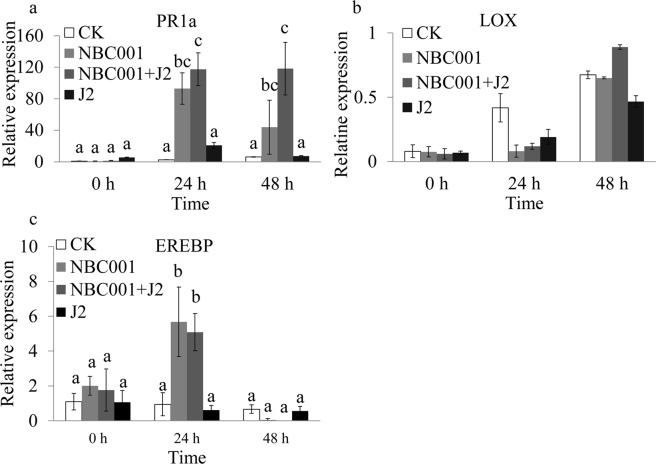
Expression of plant defense genes in soybean roots in different treatments after *H*. *glycines* infection at different time intervals. (**a**) *GmPR1a*, (**b**) *GmLOX*, and (**c**) *GmEREBP*. The data shown represent the average values from three independent experiments. The different letters on each bar represent significant differences at the 0.05 level based on Tukey’s multiple comparison test.

To determine the signaling pathways elicited by NBC001, the expression of *GmPR1a*, *GmLOX* and *GmEREBP* as SA-responsive, JA-responsive and ET-responsive marker genes, respectively, was analyzed at 0, 24 and 48 h post-inoculation using real-time quantitative PCR. The increased expression of *GmPR1a*, *GmLOX* and *GmEREBP* significantly enhanced plant defense^19^. At 24 and 48 h post-inoculation, the expression of *GmPR1a* and *GmEREBP* in the roots treated with NBC001 or NBC001 with J2 appeared to be significantly higher than that in either the roots treated with J2 or the control (Fig. 8a,c, *P* < 0.05). However, *GmLOX* was constantly expressed at a low level and did not show any differences among any of the treatment (Fig. 8b, *P* > 0.05). The increased expression of *GmPR1a* and *GmEREBP*, typical SA- or ET-responsive marker gene, suggests that the ISR response induced by NBC001 was dependent on both SA and ET signal pathways. These data further confirm that NBC001 can activate the soybean defense responses.

### Effect of NBC001 on the soil microbial communities

**Figure 9:**
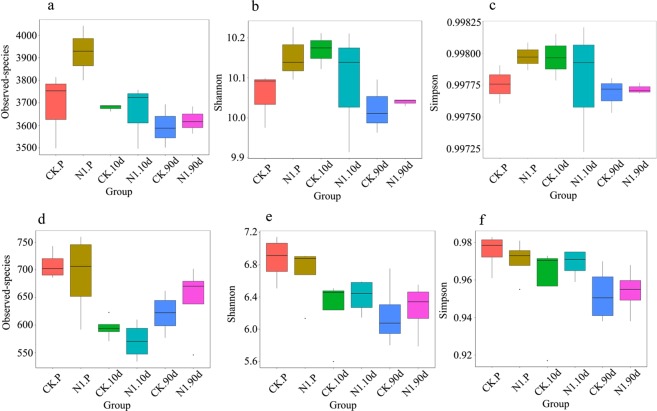
Diversity of soybean rhizosphere microorganisms in the different treatments. The numbers of observed species and the Shannon and Simpson indices were calculated from bacterial (**a–c**) and fungal (**d–f**) OTUs of soybean rhizosphere microorganisms. CK, control treatment. N1, NBC001 treatment. P, before transplantation. 10 d and 90 d, 10 and 90 days post-ransplantation, respectively.

**Figure 10:**
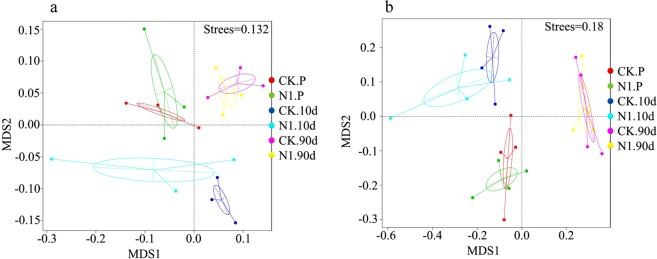
NMDS ordination plots for the soybean rhizosphere bacterial (**a**) and fungal (**b**) communities in the different treatments. The proximity between the samples in the plot corresponds to high community similarity. CK, control treatment. N, NBC001 treatment. P, before transplantation. 10 d and 30 d, 10 and 30 days post-transplantation, respectively.

Bacterial and fungal microbial richness and diversity are shown in Fig. 9. Regarding bacteria and fungi, the observed species, Shannon and Simpson indices were not significantly different in the NBC001 treatment and the control before transplantation, or at 10 and 90 d post-transplantation. Nonmetric multidimensional scaling (NMDS) analysis showed that the bacterial and fungal communities changed along with different soybean growth stages, but the bacterial and fungal communities were similar between the NBC001 treatment and the control (Fig. 10). These results suggested that the application of NBC001 has no significant impacts on the soil microbial communities.

## Discussion

*H*. *glycines* is one of the most economically important pathogens of soybean and is difficult to control. The management of *H*. *glycines* with biocontrol microorganisms is an eco-friendly method. In this study, we isolated a fungal strain, NBC001, from cysts of *Heterodera* spp. The strain NBC001 was identified as an *A*. *niger* species and found to induce the resistance of soybean against *H*. *glycines*.

*A*. *niger* is very common in all types of soils, and it has been found to effectively suppress plant parasitic nematodes and enhance the yield of plants^11^. In the greenhouse, the application of *A*. *niger* significantly reduced the soil populations of *M*. *javanica* and root galling and increased the plant height and fresh weight of mungbean shoots^20^. In the field, *A*. *niger* PD-42 significantly reduced tomato and pepper root galling due to *Meloidogyne incognita*. In this study, *H*. *glycines* in the soybean seedlings dressed with the culture filtrate of *A*. *niger* NBC001 was controlled in both the pot and field experiments.

In this study, we tested the effects of the culture filtrate of *A*. *niger* NBC001 on the J2s mortality and egg hatchability of *H*. *glycines in vitro*. The results showed that the culture filtrate of NBC001 caused 89% mortality of J2s and inhibited more than 98% of the eggs from hatching. These results indicate that NBC001 showed high nematicidal activity against *H*. *glycines*. In addition, the J2s mortality and inhibition of hatchability decreased as the dilution factor of NBC001 increased. This result indicated that NBC001 might produce nematicidal metabolites to suppress *H*. *glycines*. When the dilution factor of NBC001 was increased, the concentration of the nematicidal metabolites was reduced. Therefore, the nematicidal activity decreased. *A*. *niger* has been reported to produce toxic microbial exometabolites to kill plant parasitic nematodes^21,22^. The nematicidal metabolites in the *A*. *niger* NBC001 culture filtrate is being investigated.

In addition to direct antagonism, some biocontrol microorganisms can protect plants indirectly via the stimulation of inducible defense responses that render the hosts more resistant to further pathogen ingression^23^. Such an induction of enhanced defensive capacity, which is known as ISR, can be systemic and is effective against a broad range of diseases. These mechanisms include the reinforcement of plant cell walls, the production of antimicrobial phytoalexins and the synthesis of PR proteins^24^. However, the knowledge that biocontrol agents induced systemic resistance towards plant parasitic nematodes is rare^25^. Munif *et al*. (2001) reported that induced systemic resistance is considered a possible control mechanism of four endophytic bacteria, including *Pantoea agglomerans* MK-29, *Cedeca davisae* MK-30, *Enterobacter* spp. MK-42 and *Pseudomonas putida* MT-19, against root-knot nematodes^26^. The ability of the fungal inoculants to stimulate ISR against the nematodes was demonstrated^27^. Vu *et al*. (2006) found that *Fusarium oxysporum* control *R*. *similis* by inducing the systemic resistance of banana^28^.

Recently, Li *et al*. (2011) found that *A*. *niger* could reduce the nematode populations and promote the tomato plant growth by increasing the activities of defense enzymes^12^. These findings indicate that *A*. *niger* could induce the resistance of tomato to plant-parasitic nematodes. Therefore, in this study, we tested whether the *A*. *niger* NBC001 filtrate could activate soybean defense responses at the cellular and molecular levels. The rapid release of ROS, superoxide dismutase and hydrogen peroxide is ubiquitous in the early responses following the recognition of microorganisms^29^. ROS has direct antimicrobial activity and mediates other defense responses, including the oxidative crosslinking of plant cell walls, callose deposition and hypersensitive cell death^30^. Cell walls may be reinforced via the deposition of lignin, phenolic compounds, suberin and callose^29^. In this study, the application of *A*. *niger* NBC001 stimulated hydrogen peroxide activity and callose deposition, suggesting that *A*. *niger* NBC001 activated the cellular defenses of plant cells, thus contributing to the resistance to *H*. *glycines*. In addition, soybean seedlings dressed with the culture filtrate of *A*. *niger* NBC001 exhibited increased callose deposition in the leaves, demonstrating that NBC001 was able to activate plant induced systemic resistance against *H*. *glycines*.

Nematode infestation could activate plant defense responses, including the release of ROS, antimicrobial secondary metabolites, hydrolytic enzymes, and local fortifications of plant cell walls by lignification and callose deposits^31^. In this study, inoculation of *H*. *glycines* J2s stimulated the hydrogen peroxide activity and callose deposition in soybean. Moreover, the ROS and callose deposit area were increased in roots treated with both NBC001 and J2 compared to those in the NBC001 and J2 alone treatments. These results indicate that activation of plant defense responses by NBC001 and J2 was cooperative rather than antagonistic.

Biocontrol microorganisms commonly activate an ISR response in plants via the jasmonate (JA) and/or ethylene (ET) signaling pathways^32^. However, there are examples of SA-dependent defense responses^19,32^. In this study, we tested three genes related to the typical signaling pathways and found that the SA-regulated defense-related gene *GmPR1a* and the ET-regulated defense-related gene *GmEREBP* were activated by NBC001. However, the JA-regulated defense-related gene *GmLOX* did not show any significant differences in the roots treated with PDA and with NBC001 combined with *H*. *glycines* infection. This result indicates that the disease resistance induced by NBC001 may be controlled by the SA- and ET-dependent signaling pathways. The results are consistent with the point that the SA- and JA/ET- signaling pathways in plant protection provided by biocontrol microorganisms are cooperative rather than antagonistic^33–35^. The induction of ISR by the rhizobacteria N11.37 in *Arabidopsis thaliana* against *Xanthomonas campestris* is SA- and ET- dependent^36^.

Introduced biocontrol agents may have a short- or long-term effect on the soil microbial community^37^. In this study, we did not observe any definable risk for the soil microbe communities arising from the application of NBC001. Some researchers also found no effects on the soil microbial communities following inoculation with biocontrol agents, such as *Pseudomonas fluorescens* 2P24, *P*. *fluorescens* CPF10 and *Bacillus subtilis* Jdm2^38–40^.

These results indicate that *A*. *niger* NBC001 has potential as a commercial biological control agent to control *H*. *glycines*. Aspergillus spp. are known to be opportunistic pathogens of human beings^41^. A. fumigatus is the most common mold pathogen of human beings and can cause both invasive disease in immunocompromised patients and allergic disease in patients with atopic immune systems^42^. However, to our knowledge, there are no reports on the effect of A. niger on human beings. However, it is necessary to assess the effects of NBC001 on human beings before it is used extensively.

In summary, NBC001 may be a good biocontrol agent against *H*. *glycines* via stimulation of plant host defense. In addition, the application of NBC001 has no effects on the soil microbial communities. To increase the field performance of biocontrol agents, a detailed knowledge of environmental adaptations is required.

## Materials and Methods

### Isolation of fungi

Soil samples were collected from fields in the Xuchang District, Henan, China and Østfold, Norway in 2015. Cysts were collected from the soil samples by suspending the soil in water and filtering the samples through nested 710-μm-pore and 250-μm -pore sieves^43^. The cysts were treated with 0.5% sodium hypochlorite (NaClO) for 5 min for surface disinfestation and washed three times with sterile distilled water to remove residual NaClO^44^. Subsequently, the cysts were broken open with a rubber plug to release the eggs and passed through a 200-μm -pore sieve and collected on a 30-μm -pore sieve^45^. Free eggs were smeared on Petri dishes containing potato dextrose agar (PDA; Oxoid Ltd, Basingtoke, Hampshire, England) and inoculated at 26 °C in complete darkness for 5 d^45^. Hyphae that emerged from the eggs were transferred to PDA plates for purification and identification.

### Identification of the fungi

A single spore culture was established for each isolate, and the isolates were identified by blasting the sequences of the internal transcribed spacers (ITSs) of the rDNA regions as described by Tendulkar *et al*.^46^. Amplification of the fungal ITS fragment was performed using a PCR approach with the primers pairs ITS4 (5′-TCCTCCGCTTATTGATATGC-3′) and ITS5 (5′-GGAAGTAAAAGTCGTAACAAGG-3′)^47^.

### Nematode and fungus culture

Cysts of *H*. *glycines* were collected from Langfang, Hebei, China using the method described above. The J2s of *H*. *glycines* were obtained by incubating the cysts on a sieve (30-μm pore size) in 3 mM ZnCl_2_ at 26 °C^48^. The hatched J2s were collected daily.

The isolated fungi were cultured on a potato dextrose agar (PDA; Shanghai Bioway Technology Co., Ltd, Shanghai, China) plate and inoculated at 26 °C in complete darkness^45,49^. After 10 d, a 5-mm diameter mycelial plug of each tested fungus was inoculated into 200 mL of PDB in a 500 mL Erlenmeyer flask and incubated at 26 °C for 120 h at 150 r/min.

The culture filtrate of NBC001 was prepared by centrifuging at 2,000 rpm for 20 min^50^. Ten ml of the supernatant was filtered through a sterile 0.22 μm polyethersulfone filter (Whatman, Clifton, NJ, USA) for the *in vitro* experiments^51^. The other 10 L of supernatant was concentrated to 2 L using a vacuum freeze drier (Hitachi ES-2030) for the pot and field experiments.

### Antagonistic effect experiments

The antagonistic effects of the culture filtrate of NBC001 on the J2s and hatchability of *H*. *glycines* were performed as described by Jin *et al*.^51^. Each treatment had three replicates, and each experiment was repeated twice.

### Pot experiments

The experiment was conducted in a controlled environmental chamber with a 16 h photoperiod and a 26/20 °C day/night regimen with the soybean (*Glycine max* L. Merr., *cv*. Zhonghuang NO.13, susceptible to *H*. *glycines*). Soybean seeds were dressed with the 5-fold concentrated culture filtrate of NBC001, 5-fold concentrated PDB medium or 1.8% abamectin EC for 2 min. One soybean seed was sown in a 10-cm-diameter plastic pot filled with 1,600 cm^3^ of soil containing 280 cysts collected from fields in Langfang, Hebei, China. The treatment was conducted in a randomized complete block design (RCBD). Each treatment had ten pots and the experiment was repeated twice.

At 30 d post-transplantation, plant and rhizosphere soil samples were collected from each treatment. Cysts of *H*. *glycines* in the soil were extracted as described above. The recovered cysts were counted using a stereoscopic microscope (SZ61, Olympus Corp., Japan). The stem and root lengths were measured using a meter ruler.

### Field experiments

Field experiments were conducted in a field naturally infested with *H*. *glycines* in Langfang, Hebei, China during the 2017 cropping season. Zhonghuang NO.13 seeds were transplanted into the field after being dressed with the culture filtrate of NBC001, PDB medium or 1.8% abamectin EC for 2 min. The treatments were arranged as a randomized complete block design (RCBD) with four blocks for each treatment.

At 90 d post-transplantation, the rhizosphere soil samples were collected from each treatment. Cysts of *H*. *glycines* in the soil were extracted using the method decreased above. The yield was recorded at 120 days post-transplantation.

### Juvenile development analysis

The experiment was conducted as described above. Soybean seeds were dressed with the culture filtrate of NBC001 or PDB medium. One soybean seed was sown in a pot containing clay and sand at a 7:3 ratio (v/v). When the second leaf had emerged, 1,000 J2s of *H*. *glycines* were inoculated around the roots. The treatment was laid out in a randomized complete block design (RCBD), and each treatment had ten pots.

To observe the development of *H*. *glycines* in soybean roots, the roots were stained as described by Bybd *et al*. (1983) at 3, 7, and 14 d post inoculation^52^. After staining, juvenile at different developmental stages were examined for morphology and counted using a Leica dissecting microscope (Leica M165C, Wetzlar, Germany). Five replicates were performed for each treatment, and each assay was performed three times.

### ISR assays

A pot experiment was conducted as described above. Twenty roots and mature leaves were sampled for callose and reactive oxygen species (ROS) detection at 24 h post-inoculation. This experiment was conducted with three independent biological replicates.

Callose deposition was detected using the aniline blue staining method^53^. The stained leaves and roots were visualized using an Olympus IX83 microscope with a UV filter (Olympus Corporation, Tokyo, Japan; excitation filter 350/50 nm, replicated DM 400 dichroic beam splitter and emission filter 460/50 nm). ImagePro 5.0 software (Media Cybernetics, Bethesda, MD) was used to convert the colored micrographs to binary, and pixel densitometry was performed across the 20 images from each treatment.

ROS production was examined with the oxidant-sensitive probe dichlorodihydrofluorescein diacetate (H2DCFDA; Invitrogen, California, USA)^54^. The samples were imaged with a Carl Zeiss confocal laser scanning microscope (LSM 880; Carl Zeiss, Oberkochen, Germany).

### Real-time quantitative PCR

To test whether NBC001 could activate soybean plant defenses, we characterized the expression of the defense genes *GmPR1a*, *GmLOX* and *GmEREBP* during *H*. *glycines* infection in the presence or absence of NBC001 using real-time quantitative PCR. The soybeans were treated as described above. Soybean roots were collected at 0, 24, and 48 h post-inoculation. Total RNA was extracted from the soybean roots using TRIzol reagent (Invitrogen, Carlsbad, CA). The total RNA was treated with recombinant DNase I (Takara, Shiga, Japan) and the recombinant RNase inhibitor (Takara, Shiga, Japan) according to the manufacturer’s instructions. The DNase-treated RNA (1 μg) was used for cDNA synthesis, which was performed using the M-MLV Reverse Transcriptase (Takara, Shiga, Japan) according to the manufacturer’s instructions. The cDNA template was amplified by real-time quantitative PCR using the SYBR® Premix Dimmer Eraser kit (Takara, Shiga, Japan) according to the manufacturer’s instructions, and the PCRs were performed on an ABI 7500 Real-Time PCR System (Applied Biosystems). The primers are listed in Table 3. *Actin* was used as an internal control. The results were analyzed using SDS 2.0 software (Applied BioSystems), and the gene expression changes were calculated using the 2^−△△CT^ method.

**Table 3:** Sequences of the gene-specific primers used for real-time quantitative PCR.

Gene	Forward/reverse primers	Reference
*GmActin*	F: 5′-GAGCTATGAATTGCCTGATGG-3′ R: 5′-CGTTTCATGAATTCCAGTAGC-3′	^55^
*GmPR1a*	F: 5′-GGGTGATGTTGCCTACGCTCAA-3′ R: 5′-CAGCAACCGTATCATCCCAAGC-3′	^19^
*GmLOX*	F: 5′-TGGAGGTTTTAAGAGGAGATGG-3′ R: 5′-CCTGCGAGGGTAAGGATAGTTG-3′	^19^
*GmEREBP*	F: 5′-GATTACTCCCACATCGCTACCC-3′ R: 5′-AGATTCTTCCTCTGCCTCTTCA-3′	^19^

### Soil microbial community analysis

Field experiments were conducted as described above. Rhizosphere soil samples were collected from each plot immediately before treatment and at 10 and 90 days post-transplantation.

Total DNA was extracted from 0.5 g soil samples using the PowerSoil DNA isolation kit (Mo Bio Laboratories, Inc., Carlsbad, CA) according to the manufacturer’s instructions. The DNA extracted from each soil sample served as a template for the 16 S rRNA gene and ITS region amplification. The bacterium-biased primers 515 F/806 R and fungal-specific primers 1737F/2043 R were used. All the PCRs were performed using a Phusion® High-Fidelity PCR Master Mix (New England Biolabs). Sequencing libraries were conducted on the Ion S5TM XL platform (Life Technologies, USA, Novogene Bioinformatics Technology Co., Ltd, Beijing, China).

### Data analyses

Alpha diversity was applied to analyze the complexity of the species diversity in each sample using three indices, including observed species, Shannon and Simpson. The community structure was analyzed using nonmetric multidimensional scaling (nMDS). They were calculated using QIIME (Version1.7.0) and displayed with R software (Version 2.15.3).

All other data were analyzed using SPSS 15.0 (SPSS Inc., IBM, USA). One-way analysis of variance (ANOVA) and Tukey’s multiple comparison test were conducted to test for significant differences among the treatments. All statistical tests were performed at a significance level of 0.05.

## Data Availability

The datasets analyzed during the present study are available from the corresponding author upon reasonable request.
